# Procedural Predictors for Bioresorbable Vascular Scaffold Thrombosis: Analysis of the Individual Components of the “PSP” Technique

**DOI:** 10.3390/jcm8010093

**Published:** 2019-01-15

**Authors:** Zisis Dimitriadis, Alberto Polimeni, Remzi Anadol, Martin Geyer, Melissa Weissner, Helen Ullrich, Thomas Münzel, Tommaso Gori

**Affiliations:** 1 Zentrum für Kardiologie, University Hospital Mainz, 55131 Mainz, Germany; zisis.dimitriadis@unimedizin-mainz.de (Z.D.); polimeni@unicz.it (A.P.); remzi.anadol@unimedizin-mainz.de (R.A.); martin.geyer@unimedizin-mainz.de (M.G.); melissaweissner@web.de (M.W.); hullrich@students.uni-mainz.de (H.U.); tmuenzel@uni-mainz.de (T.M.); 2 German Center for Cardiac and Vascular Research (DZHK), Standort Rhein-Main, 55131 Mainz, Germany; 3 Division of Cardiology, Department of Medical and Surgical Sciences, Magna Graecia University, 88100 Catanzaro, Italy

**Keywords:** PSP, implantation technique, bioresorbable scaffold

## Abstract

The technique used at the time of implantation has a central role in determining the risk of thrombosis in bioresorbable vascular scaffolds (BRS). Different definitions of the “optimal” implantation technique exist, however. The impact of individual procedural characteristics on the risk of scaffold thrombosis (ScT) was evaluated in a single-center observational study that enrolled 657 patients (79% males, mean age 63 ± 12 years) with 763 lesions who received a total of 925 BRS for de novo lesions. During a median 1076 (762–1206) days’ follow-up there were 28 cases of thrombosis. Independent predictors of ScT included the use of predilatation balloons bigger than the nominal BRS diameter (hazard ratio (HR) = 0.4 (0.16–0.98), *p* = 0.04), sizing (implantation in vessels with reference vessel diameter >3.5 mm or <2.5 mm: HR = 5.71 (2.32–14.05), *p* = 0.0002) and the degree of vessel expansion (ratio of minimum lumen to reference vessel diameter, HR: 0.005 (0.0001–0.23), *p* = 0.007). In addition, a mild BRS oversizing (final BRS diameter to vessel diameter 1.14–1.28) was associated with a lower thrombosis risk, whereas undersizing and more severe oversizing (final BRS diameter to vessel diameter <1.04 and >1.35, respectively) were associated with an increased risk of ScT (HR = 0.13 (0.02–0.59), *p* = 0.0007). In conclusion, different components of the “optimal” technique have different impacts on the risk of BRS thrombosis. Besides predilatation with a balloon larger than the BRS diameter, correct vessel size selection and a mild to moderate oversizing appear to be protective.

## 1. Introduction

Though safe and effective, implantation of metallic drug eluting stents remains associated with a yearly incidence of cardiac adverse events (cardiac death, myocardial infarction, and target vessel revascularization) in the range of 1.5–3% [1,2,3]. Since risk is possibly (at least in part) to be attributed to the permanent nature of these devices [4], the introduction of bioresorbable vascular scaffolds (BRS) was expected to have favorable long-term effects by allowing vessel remodeling and the return of vasomotion. Despite initial promising reports [5,6], however, several randomized controlled studies and meta-analyses with long-term follow-up consistently reported a significantly increased risk of device thrombosis (ScT) as compared to newer generation metallic stents [7,8,9,10,11,12], which led to the removal of these devices from the market. Initial studies indicated that the increased ScT risk was due to a higher strut thickness of BRS as compared to metallic stents, which can disrupt blood´s laminar flow, eventually leading to fibrin deposition [13,14,15]. 

A number of studies have shown that the implantation technique used significantly reduces the incidence of early but also late events [16,17,18,19,20,21,22,23]. Accordingly, different sets of recommendations for BRS implantation were issued, the most recent one being known as ‘PSP’: pre-dilatation (lesion preparation), correct sizing of the BRS, and post-dilatation [17,24,25]. Of note, while this implantation technique has been shown to reduce risk, it remains to be analyzed which of the components of this technique are associated with this improvement in the patient’s prognosis. Finally, the aim of the present study was to evaluate the impact of individual procedural characteristics on the risk of scaffold thrombosis in patients who have undergone BRS implantation.

## 2. Methods

### 2.1. Objectives and Design

The aim of the study was to evaluate the different individual components of the implantation technique and their impact on the incidence of ScT and target lesion failure (defined as the composite of cardiovascular death, target vessel myocardial infarction, and target lesion revascularization). The clinical characteristics and quantitative coronary analysis (QCA) data of all patients treated with a BRS (Absorb 1.1, Abbott Vascular, Santa Clara, CA, USA) between May 2012 and January 2015 were collected in a single-center retrospective registry.

The study belongs to the MICAT program, which is approved by the ethics committee of the Landesärztekammer Mainz. ClinicalTrials.gov Identifier: NCT02180178). The incidence, clinical presentation, and predictors of ScT in this set of patients, as well as more details on the data collection procedures, have already been reported [26].

### 2.2. Use of BRS and Definitions

Mechanical limitations restrict the use of BRS. As such, BRS were not implanted (and therefore the present data do not apply) in the following situations: bypass grafts, in-stent restenosis, severely calcific or tortuous vessels, and bifurcations with side branch >2 mm. Dual antiplatelet therapy (with prasugrel or ticagrelor for acute coronary syndrome patients) was recommended for 12 months in all patients. A description of the definitions used for the diagnosis and timing of ScT (as based on the Academic Research Consortium criteria [27]) is reported in [16]. The adjudication of events was based on a review of the original clinical data by two investigators not involved on other analyses.

### 2.3. Quantitative Coronary Analysis

Methods, definitions, measurements performed, and local reproducibility are described in detail in the online supplement. Parameters refer to measurements performed before and immediately after implantation. Key measures included:

- MLD: in-BRS minimum lumen diameter.
- RVD: interpolated reference vessel diameter.

Derived parameters include the following:

- BRS nominal size divided by RVD.
- Postprocedural residual stenosis (RVD minus MLD) divided by RVD.
- Postprocedural MLD/RVD ratio.
- Postprocedural MLD/nominal, footprint and scaled residual stenosis (markers of device expansion).

These parameters are described in detail in the online supplement.

The QCA analysis was performed by staff blinded to the clinical characteristics and outcome of the patients.

### 2.4. Implantation Technique

The following individual parameters were analyzed with respect to their impact on ScT:

- Reference vessel diameter (RVD) <2.5, comprised between 2.5 and 3.5 or >3.5 mm,
- Ratio of predilatation balloon diameter to reference vessel diameter,
- Ratio of predilation balloon diameter to nominal BRS diameter,
- Ratio of true BRS diameter to RVD,
- BRS implantation at high pressure (>12 atmospheres (ATM)),
- BRS implantation at very high pressure (>16 ATM),
- Use of post-dilatation,
- Ratio of post-dilatation balloon diameter to BRS nominal diameter,
- Post-dilatation with high pressure (>12 ATM),
- Post-dilatation with very high pressure (>16 ATM).

In order to determine whether optimal implantation (independently of the technique used) was associated with the endpoints of interest, we analyzed the predictive role of the following parameters:

- Ratio of postprocedural minimum lumen diameter (MLD) to RVD (a marker of vessel expansion),
- Ratio of postprocedural MLD to nominal BRS diameter (a marker of BRS expansion).

Finally, we used the inflation charts to calculate the final BRS diameter and evaluate the ratio between this parameter and the RVD at 0.1 percentile intervals.

### 2.5. Statistical Analysis

Continuous variables are presented as mean ± standard deviation (SD) or median (interquartile range) based on the inspection of the Q‒Q plots. Accordingly, they were compared using Student’s *t*, Mann-Whitney, Kruskall-Wallis, or analysis of variance. Categorical variables are presented as counts and percentages, and were compared using the Fisher’s exact test.

Using univariate Cox regression analysis, we tested the effect of each of the following parameters on ScT and target lesion failure (TLF):

- Predilatation with balloon diameter smaller than nominal vessel diameter,
- Predilatation with balloon diameter smaller than RVD,
- RVD <2.5 mm or >3.5 mm,
- Post-dilatation,
- Post-dilatation with a balloon larger than nominal diameter,
- Post-dilatation with high pressure (>12 ATM),
- Post-dilatation with very high pressure (>16 ATM),
- True BRS diameter larger than RVD,
- MLD/RVD,
- MLD/nominal and residual stenosis.

Parameters with a *p* < 0.1 were included in the multivariate analysis. Separate analyses were also performed for subacute and late ScT.

The correlation coefficient was used to describe the association between each procedural parameter and the final MLD/RVD ratio.

Kaplan-Meier curves were built to derive the event rates and plot time-to-event curves.

Cox proportional hazards analysis was performed to identify the clinical and procedural parameters relevant for the ScT separately. Univariate and multivariable hazard ratios (HRs) and 95% confidence intervals (CIs) are calculated accordingly. Statistical tests and analyses were performed with Medcalc (Version 9, Mariakerke, Belgium).

## 3. Results

As previously published, the database included 657 patients (mean age of 63 ± 12 years, 79% males, 22% diabetics, 63% acute coronary syndromes) who received a total of 925 BRS (1.4 ± 0.8 per patient), with a median follow-up of 1076 (762–1206) days. The clinical and procedural characteristics of the patients, as well as the clinical presentation, sequelae and predictors of ScT, have been published elsewhere (20). Overall, 25 definite, two probable, and one possible ScT occurred at a median of 69 (0–451 days, and 90 TLF occurred at a median of 346 (64–609) days after implantation. 

### 3.1. The Impact of Procedural Parameters on ScT

The results of the Cox regression analysis are presented in Supplementary Table S1 and Table 1. The procedural parameters independently associated with ScT included vessel size <2.5 (4.63 (1.78–12.05)) or >3.5 mm (HR = 4.1 (1.49–11.30)). The ratio of MLD to RVD, expressing vessel expansion, was also associated with incident ScT. 

RVD <2.5 mm remained a predictor of acute ScT (HR = 4.77 (1.63–13.94)), while RVD>3.5 was a predictor of late ScT (HR = 2.86 (1.31–6.27)). A low ratio of MLD to nominal BRS diameter, expressing incomplete BRS expansion, was associated with early ScT (HR = 0.0003 (0–0.7); in contrast, a low ratio MLD to RVD, expressing incomplete vessel expansion, was associated with late ScT (HR = 0.003 (0–0.11)).

**Figure 1:**
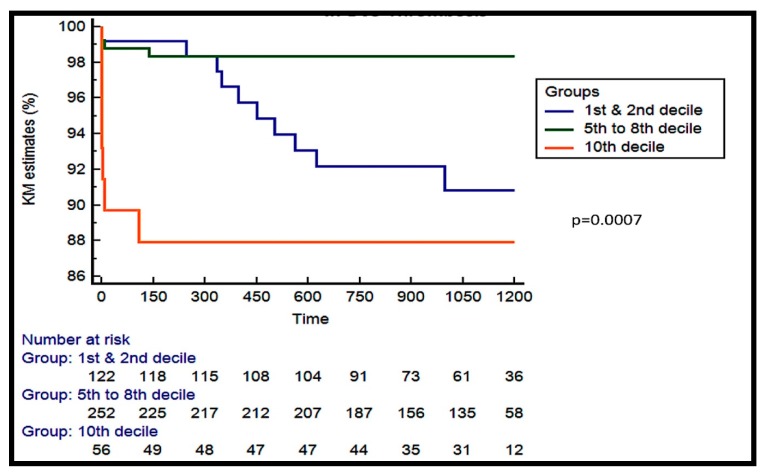
Kaplan-Meier analysis of the true final BRS diameter to RVD divided in 0.1 percentile interval with respect to scaffold thrombosis.

To investigate further the role of BRS sizing (ratio of final BRS size to RVD), we analyzed the association between the ratio between true final BRS diameter and reference vessel diameter in 0.1 percentile intervals. The analysis showed that mild BRS oversizing (final BRS diameter to vessel diameter 1.14–1.28) correlates with a lower thrombosis risk. In contrast, more severe oversizing (final BRS diameter to vessel diameter >1.35) or undersizing (final BRS diameter to vessel diameter <1.04) was associated with increased thrombosis risk (*p* = 0.0007, HR = 0.13, 95% CI 0.02–0.59, Figure 1).

### 3.2. The Impact of Procedural Parameters on TLF

The results of the cox regression analysis are presented in Supplementary Table S2 and Table 2. The procedural parameters independently associated with TLF included vessel size <2.5 (1.89 (1.18–3.07)) and post-dilatation with very high pressure (HR = 0.41 (0.18–0.97)). The ratio of MLD to RVD, expressing vessel expansion, was also associated with incident TLF (HR = 0.004 (0.00–0.04)). 

### 3.3. The Impact of Procedural Parameters on the Ratio of MLD to RVD

Given its importance as a marker of subsequent risk of late/very late ScT and TLF, we investigated the relationship between procedural parameters and MLD/RVD (Table 3). Notably, none of the parameters was associated with more (higher) favorable MLD/RVD. Use of post-dilatation balloons larger than the nominal BRS diameters was actually associated with a paradoxically lower MLD/RVD.

## 4. Discussion

The initial enthusiasm about BRS has been tempered by various studies and meta-analyses reporting a higher risk of device thrombosis as compared to newer generation drug-eluting stents [8,11]. As recently demonstrated by various research groups, however, this increased risk may be modulated with an appropriate implantation technique [16,17]. In line with this, a set of recommendations, including predilation, 1:1 sizing, and high-pressure post-dilatation was developed. These guidelines have, however, been introduced in toto, and it remains unclear which of the recommended procedural steps has an impact on patients’ prognosis. The present study evaluated the impact of the individual procedural parameters in a real-world cohort of patients treated with BRS. 

We found important differences in the procedural predictors of different outcome. Importantly, none of the operator-dependent parameters (size of the predilation or post-dilatation balloon, implantation pressure, post-dilatation pressure) had an impact on the incidence of ScT. In contrast, strong relationships were shown between vessel size and incident ScT. In particular, early ScT was associated with a vessel size smaller than 2.5 mm, while late/very late ScT was associated with RVDs larger than 3.5 mm. This important finding was also reflected by the associations between the postprocedural parameters and ScT. While the ratio between MLD/nominal BRS diameter was associated with early ScT, the ratio of MLD/RVD was associated with late/very late ScT. These findings likely reflect the different mechanisms underlying ScT at different timepoints: while the prevailing mechanism of early ScT is incomplete BRS expansion, which results in increased footprint (see [16,28] for a discussion), late/very late is determined by incomplete expansion of stenoses in large vessels, resulting at the same time in flow disturbances due to the post-stenotic flow separation and the strut malapposition [29]. Finally, we found that mild oversizing (true BRS/RVD between 1.14 and 1.28) was associated with the lowest overall ScT risk, while more severe oversizing (true BRS/RVD > 1.35) and undersizing (true BRS/RVD between 0.97 and 1.04) showed significantly higher ScT.

When the possible determinants of MLD/RVD were investigated, we found that none of the procedural factor had an impact on this parameter. Notably, the use of post-dilatation balloons larger than the nominal BRS diameters was associated with a paradoxically lower MLD/RVD. Although the observational nature of the present data does not allow for making inferences, it could be hypothesized that an excessively aggressive BRS overexpansion might have led to an increased incidence of strut fractures and recoil [30]. In line with this, Yamaji et al. observed that high-pressure post-dilatation yields a small improvement in in-device minimal lumen area, while being associated with significantly larger angiographic late lumen loss at six months [31]. Recently, many studies reported that short-duration dual antiplatelet therapy (DAPT) (≤6 months) demonstrated a similar incidence of net adverse cardiovascular and clinical events at 12 months in ACS compared with the standard duration DAPT (≥12 months) after second-generation drug eluting stent implantation [32]. However, it would be reasonable to prolong the duration of DAPT for more than 12 months to reduce the risk of ScT in patients who have undergone BRS implantation because of the augmented risk of ScT.

The observational nature of the present data has intrinsic limitations. Although the methods used for the acquisition and analysis of the data were analogous to those used in randomized controlled trials, the lack of randomization, some differences in baseline characteristics, and the operator-dependent observations do not allow mechanistic conclusions, so the evidence provided here should be considered as hypothesis-generating and exploratory. However, the absence of inclusion/exclusion criteria and the complexity of the patients/lesions treated here provide information that is more directly applicable. 

## 5. Conclusions

Whatever the mechanism, the present data appear to suggest that no action taken by the operator, except for the choice of vessels sized between 2.5 and 3.5 mm, affects the incidence of ScT.

## Figures and Tables

**Table 1:** Multivariate Cox regression analysis of the individual procedural and postprocedural characteristics with respect to scaffold thrombosis.

Procedural Parameters	*p*	Odds Ratio	95% CI
Predilation with balloon >nominal diameter	0.08	0.47	0.20–1.10
RVD < 2.5	0.002	4.63	1.78–12.05
RVD > 3.5	0.007	4.10	1.49–11.30
Postprocedural parameters			
MLD/RVD	0.007	0.005	0.0001–0.23
MLD/Nominal	0.7	0.52	0.01–18.89
	acute or subacute In-Scaffold Thrombosis		
Procedural parameters	*p*	Odds Ratio	95% CI
RVD < 2.5 mm	0.005	4.77	1.63–13.94
Postprocedural parameters	*p*	Odds Ratio	95% CI
MLD/RVD	0.83	1.78	0.009–358.34
MLD/Nominal	0.004	0.0003	0–0.07
	late or very late In-Scaffold Thrombosis		
Procedural parameters	*p*	Odds Ratio	95% CI
Predilation with balloon >nominal diameter	0.10	0.51	0.23–1.14
RVD > 3.5	<0.0001	2.86	1.31–6.27
Postprocedural parameters			
MLD/RVD	0.002	0.003	0.0001–0.11

BRS: bioresorbable scaffolds; ATM: atmospheres; CI: confidence interval; MLD: minimum lumen diameter; RVD: reference vessel diameter.

**Table 2:** Multivariate Cox regression analysis of the individual procedural and postprocedural characteristics with respect to TLF.

Procedural Parameters	*p*	Odds Ratio	95% CI
Post-dilatation in all BRS	0.179	0.73	0.46–1.15
RVD < 2.5	0.009	1.89	1.18–3.07
Post-dilatation with high pressure (>16 ATM)	0.04	0.41	0.18–0.97
Postprocedural parameters			
MLD/Nominal	0.14	0.21	0.03–1.61
MLD/RVD	<0.0001	0.004	0.00–0.04

BRS: bioresorbable scaffolds; ATM: atmospheres; CI: confidence interval; MLD: minimum lumen diameter; RVD: reference vessel diameter.

**Table 3:** Median (IQR) and correlation coefficient for evaluation of the impact of different procedural predictors with respect to the MLD/RVD ratio.

	MLD/RVD (Median and IQR)	Corr. Coeff ± SE *p*
Predilation with balloon ≤nominal	0.88 (0.82–0.93)	−0.007 ± 0.009 0.4
Predilation with balloon >nominal	0.87 (0.8–0.93)	
Predilation with balloon ≤RVD	0.87 (0.8–0.92)	0.02 ± 0.01 0.1
Predilation with balloon >RVD	0.89 (0.83–0.94)	
Post-dilatation ≤ nominal BRS diameter	0.88 (0.82–0.93)	−0.03 ± 0.01 0.003
Post-dilatation > nominal BRS diameter	0.84 (0.76–0.93)	
Post-dilatation without high pressure (<12 ATM)	0.87 (0.8–0.92)	0.01 ± 0.01 0.9
Post-dilatation with high pressure (>12 ATM)	0.88 (0.81–0.93)	
Post-dilatation without high pressure (<16 ATM)	0.88(0.8–0.93)	0.002 ± 0.01 0.9
Post-dilatation with high pressure (>16 ATM)	0.88 (0.81–0.93)	

ATM: atmospheres; IQR: interquartile range; BRS: bioresorbable scaffolds; MLD: minimum lumen diameter; RVD: reference vessel diameter; SE: standard error.

## Supplementary Materials

The following are available online at http://www.mdpi.com/2077-0383/8/1/93/s1, Table S1: Univariate Cox regression analysis of the individual procedural and postprocedural characteristics with respect to scaffold-thrombosis, Table S2: Univariate Cox regression analysis of the individual procedural and postprocedural characteristics with respect to TLF.
