# Supplementary material for: Procedural Predictors for Bioresorbable Vascular Scaffold Thrombosis: Analysis of the Individual Components of the “PSP” Technique
Source: J Clin Med. 2019 Jan 15;8(1):93. doi: 10.3390/jcm8010093 (PMC6352132; doi:10.3390/jcm8010093)
Supplement: Supplementary file 1 [file jcm-08-00093-s001.pdf]

Supplementary Methods:

Data collection and Follow-up

Data collection was performed by trained personnel by a review of in-house clinical documents. All data were monitored by personnel not involved in primary data entry. Follow-up data were collected by clinical outpatient visit, telephone interview, contact with family physicians, or a review of clinical records from other hospitals. All patients were contacted at least five times by phone, and, if these attempts were unsuccessful, in writing. Original medical records of each event were collected. Events were adjudicated by consensus of at least two experienced cardiologists.

QCA analysis

Coronary angiography and OCT sequences were digitally recorded and analysed offline (Xcelera, Philips, the Netherlands) by specifically trained independent staff and reviewed by interventional cardiologists (1). Recently published BRS definitions were used (2). Endpoints definitions, repeatability and reproducibility data are reported elsewhere (1).

Supplementary Table S1. Univariate Cox regression analysis of the individual procedural and postprocedural characteristics with respect to scaffold thrombosis.

Univariate Analysis	In-Scaffold Thrombosis		
	P	Odds Ratio	95% CI
Predilation with balloon > nominal diameter	0.09	0.52	0.25–1.11
Predilation with balloon > RVD	0.70	0.9	0.4–1.85
RVD <2.5	**0.05**	2.25	0.99–5.08
RVD>3.5	**0.003**	3.30	1.53–7.12
Post-dilatation in all BRS	0.11	0.51	0.23–1.16
Post-dilatation > nominal	0.46	0.67	0.24–1.93
Post-dilatation with high pressure (>12 atm)	0.44	0.67	0.56–3.84
Post-dilatation with high pressure (>16 atm)	0.2	0.39	0.09–1.63

True BRS lumen > RVD	0.09	0.44	0.18–1.12
Postprocedural parameters			
MLD/RVD	**<0.0001**	0.003	0.0003–0.04
MLD/Nominal	**0.0002**	0.014	0.001–0.20
	**acute or subacute In-Scaffold Thrombosis**		
	P	Odds Ratio	95% CI
Predilation with balloon > nominal diameter	0.95	1.03	0.36–2.97
Predilation with balloon > RVD	0.21	1.98	0.69–5.69
RVD <2.5	**0.001**	5.62	1.98–15.93
RVD>3.5	0.45	0.46	0.06–3.45
Post-dilatation in all BRS	0.51	0.69	0.23–2.05
Post-dilatation > nominal	0.26	0.31	0.04–2.35
Post-dilatation with high pressure (>12 atm)	0.68	0.78	0.25–2.48
Post-dilatation with high pressure (>16 atm)	0.36	0.39	0.05–2.94
Nominal BRS diameter > RVD*1.1	0.30	1.50	0.70–3.24
Nominal BRS diameter < RVD*0.9	**0.04**	2.21	1.02–4.77
Postprocedural parameters			
MLD/RVD	**0.0019**	0.003	0.0001–0.09
MLD/Nominal	**<0.0001**	0.0004	0–0.02
	**late or very late In-Scaffold Thrombosis**		
	P	Odds Ratio	95% CI
Predilation with balloon > nominal diameter	**0.01**	0.07	0.01–0.55
Predilation with balloon > RVD	0.08	0.27	0.06–1.18
RVD < 2.5	0.42	0.43	0.06–3.27
RVD > 3.5	**<0.0001**	10.70	3.61–31.75
Post-dilatation in all BRS	0.12	0.36	0.10–1.29
Post-dilatation > nominal	0.88	1.1	0.31–3.94
Post-dilatation with high pressure (>12 atm)	0.16	4.23	0.56–32
Post-dilatation with high pressure (>16 atm)	0.36	0.39	0.05–2.94
Nominal BRS diameter > RVD*1.1	0.13	0.21	0.28–1.59
Nominal BRS diameter < RVD*0.9	**0.0004**	7.08	2.39–21.01
Postprocedural parameters			
MLD/RVD	**0.002**	0.003	0.0001–0.11
MLD/Nominal	0.9	0.8	0.01–43.15

J. Clin. Med. **2019**, *8*, 93; doi:10.3390/jcm8010093

Supplementary Table S2. Univariate Cox regression analysis of the individual procedural and postprocedural characteristics with respect to TLF.

Univariate Analysis	Target Lesion Revascularization		
	P	Odds Ratio	95% CI
Predilation with balloon > nominal diameter	0.293	0.80	0.53-1.21
Predilation with balloon > RVD	0.218	0.99	0.65-1.53
RVD < 2.5	**0.011**	1.86	1.15-3.01
RVD > 3.5	0.131	1.53	0.88-2.67
Post-dilatation in all BRS	**0.031**	0.61	0.39-0.95
Post-dilatation > nominal	0.73	0.91	0.53-1.56
Post-dilatation with high pressure (>12 atm)	0.330	0.80	0.51-1.25
Post-dilatation with high pressure (>16 atm)	**0.017**	0.36	0.16-0.83
True BRS lumen > RVD	0.274	0.73	0.42-1.27
Postprocedural parameters			
MLD/RVD	**<0.0001**	0.001	0.003-0.005
MLD/Nominal	**<0.0001**	0.175	0.08-0.39
